# Growth pattern in children with X-linked hypophosphatemia treated with burosumab and growth hormone

**DOI:** 10.1186/s13023-022-02562-9

**Published:** 2022-11-12

**Authors:** Diana-Alexandra Ertl, Justin Le Lorier, Andreas Gleiss, Séverine Trabado, Candace Bensignor, Christelle Audrain, Volha Zhukouskaya, Régis Coutant, Jugurtha Berkenou, Anya Rothenbuhler, Gabriele Haeusler, Agnès Linglart

**Affiliations:** 1 AP-HP, Reference Center for Rare Disorders of the Calcium and Phosphate Metabolism, Filière OSCAR and Platform of Expertise for Rare Diseases Paris-Sud, Bicêtre Paris-Saclay Hospital, 78 Rue du Général Leclerc, 94270 Le Kremlin Bicêtre, France; 2 University Paris Saclay, Le Kremlin-Bicêtre, France; 3 AP-HP, Department of Endocrinology and Diabetology for Children and Department of Adolescent Medicine, Bicêtre Paris-Saclay Hospital, Le Kremlin-Bicêtre, France; 4 Department of Pediatrics and Adolescent Medicine, Division of Pulmonology, Allergology and Endocrinology, Medical University of Vienna, Vienna, Austria; 5 Vienna Bone and Growth Center, Vienna, Austria; 6 Center for Medical Statistics, Informatics, and Intelligent Systems, Medical University of Vienna, Vienna, Austria; 7 Department of Molecular Genetics, Pharmacogenetics and Hormonology, Inserm U1185 and University Paris Saclay, AP-HP Bicêtre Paris-Saclay Hospital, Le Kremlin-Bicêtre, France; 8 CHU Dijon Bourgogne, Pediatric Hospital, Dijon, France; 9 Laboratory Orofacial Pathologies, Imaging and Biotherapies URP2496 and FHU-DDS-Net, Dental School, Platforme d´Imaginerie du Vivant (PIV), University Paris Cite, Montrouge, France; 10 AP-HP Cochin Hospital, Department of Diabetology, University Paris Cite, Paris, France; 11 Department of Pediatric Endocrinology and Diabetes, CHU Angers, Anger, France

**Keywords:** X-linked hypophosphatemia (XLH), Burosumab, Growth, Recombinant human growth hormone, Children

## Abstract

**Background:**

X-linked hypophosphatemia (XLH) is characterized by increased serum concentrations of fibroblast growth factor 23 (FGF23), hypophosphatemia and insufficient endogenous synthesis of calcitriol. Beside rickets, odonto- and osteomalacia, disproportionate short stature is seen in most affected individuals. Vitamin D analogs and phosphate supplements, i.e., conventional therapy, can improve growth especially when started early in life. Recombinant human growth hormone (rhGH) therapy in XLH children with short stature has positive effects, although few reports are available. Newly available treatment (burosumab) targeting increased FGF23 signaling leads to minimal improvement of growth in XLH children. So far, we lack data on the growth of XLH children treated with concomitant rhGH and burosumab therapies.

**Results:**

Thirty-six patients received burosumab for at least 1 year after switching from conventional therapy. Of these, 23 received burosumab alone, while the others continued rhGH therapy after switching to burosumab. Children treated with burosumab alone showed a minimal change in height SDS after 1 year (mean ± SD 0.0 ± 0.3 prepubertal vs. 0.1 ± 0.3 pubertal participants). In contrast, rhGH clearly improved height during the first year of treatment before initiating burosumab (mean ± SD of height gain 1.0 ± 0.4); patients continued to gain height during the year of combined burosumab and rhGH therapies (mean ± SD height gain 0.2 ± 0.1). As expected, phosphate serum levels normalized upon burosumab therapy. No change in serum calcium levels, urinary calcium excretion, or 25-OHD levels was seen, though 1,25-(OH)_2_D increased dramatically under burosumab therapy.

**Conclusion:**

To our knowledge, this is the first study on growth under concomitant rhGH and burosumab treatments. We did not observe any safety issue in this cohort of patients which is one of the largest in Europe. Our data suggest that continuing treatment with rhGH after switching from conventional therapy to burosumab, if the height prognosis is compromised, might be beneficial for the final height.

**Supplementary Information:**

The online version contains supplementary material available at 10.1186/s13023-022-02562-9.

## Background

X-linked hypophosphatemia (XLH), caused by loss-of-function variants of the *PHEX* gene, located on Xp22.11 and encoding a membrane protein expressed in bone and teeth tissues [1, 2]. Pathogenic variants in *PHEX* are associated with elevated serum concentrations of fibroblast growth factor 23 (FGF23), a molecule secreted by osteocytes and osteoblasts, which downregulating the expression of sodium-dependent phosphate transport proteins 2A and 2C, leading to a diminished reabsorption of phosphate in the proximal renal tubule, an increase in urinary elimination of phosphate, and, as a result, hypophosphatemia. It is still unclear how *PHEX* variants lead to *FGF23* overexpression. XLH is a rare disease but represents the most frequent genetic form of phosphate wasting, with an incidence of 1.5–4.8 per 100,000 [3, 4]. High levels of FGF23 also reduce kidney expression of 1-alpha-hydroxylase, and hence renal hydroxylation of vitamin D3, leading to insufficient endogenous production of calcitriol [5, 6]. Additionally, the elevated FGF23 levels increase the expression of vitamin D 24-hydroxylase, hence increasing the catabolism of both 25-hydroxyvitamin D (25-OHD) and calcitriol, which leads to reduced intestinal phosphate and calcium absorption [7, 8]. Phosphate is of vital importance for a large variety of functions, from being part of important molecules (e.g. DNA, ATP), up to influencing intra- and intercellular signaling. Most important, phosphate is a major constituent of bone and teeth, forming hydroxyapatite together with calcium, thus it has a major role in mineralization [4, 9]. Lack of proper *PHEX* function also likely leads to impaired chondrogenesis [10, 11]. The clinical presentation of XLH includes mainly rickets, osteomalacia, with a progressive and severe deformation of the lower extremities, craniosynostosis, odontomalacia and disproportionate short stature [3, 4], but the spectrum of comorbidities can be a lot brighter than this, depending on the gravity of the phenotype. So, not rarely, we do encounter patients additionally presenting with, for example, nephrolithiasis or chronic renal disease, arterial hypertension, secondary hyperparathyroidism, myopathy, or hearing loss [4].

Most patients become symptomatic in the first 2 or 3 years of life and early initiation of vitamin D analogs and phosphate supplements, i.e., conventional therapy, prevents worsening of or improves symptoms. Further, it has been shown through observational studies that height is maintained or improved in XLH children receiving conventional therapy. This effect is, however, moderate as most patients still have short stature, i.e., a final height below − 2SD, when they reach adulthood [12–15]. Recombinant human growth hormone (rhGH) has been used in XLH children with short stature in combination with conventional therapy. There is evidence in the literature that rhGH therapy increases longitudinal growth velocity without worsening body disproportion in XLH [16–18], especially in prepubertal individuals [14, 19–21]. Nonetheless, data on the final height after rhGH therapy are scarce [22–24]. Since 2018, a treatment targeting increased FGF23 signaling has been available, namely, a human monoclonal IgG antibody, burosumab, which antagonizes the actions of FGF23 [25]. In children, clinical trials and reports have shown that this therapy restores renal reabsorption of phosphate, and improves serum phosphate and circulating calcitriol. It exerts significant positive effects on rickets and bone deformities in XLH children. However, the effect of FGF23 blockade on growth velocity appears mild [25–29]. Given that height is a major clinical outcome in XLH patients, given the lack of safety and efficacy data from patients receiving concomitant treatment with burosumab and rhGH, and taking advantage of our prospective longitudinal observational cohort of XLH children and adolescents treated with burosumab, we report here the growth patterns and safety data of XLH children treated with burosumab, or treated with burosumab and rhGH.

### Study objectives

**Fig. 1:**
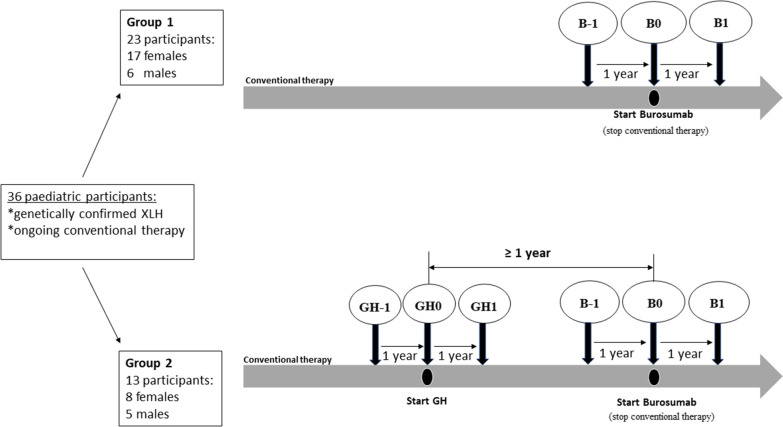
Study design and groups. B-1: 1 year before burosumab initiation, B0: initiation of burosumab therapy, B1: 1 year after burosumab initiation, GH0: initiation of rhGH therapy, GH-1: 1 year before rhGH initiation, GH1: 1 year after rhGH initiation

The primary objective of our study was to analyze the growth patterns of children affected by XLH who received burosumab after switching from the conventional therapy. In addition, we analyzed the growth response and the safety of rhGH in XLH children treated with the combination of burosumab and rhGH for at least 1 year after switching to burosumab (Fig. 1). We analyzed the ∆H SDS between and within the two groups of XLH children.

As a secondary objective, we explored biochemical changes under rhGH and/or burosumab therapy, related to the underlying condition of XLH (e.g., changes in levels of serum alkaline phosphatase and phosphate, and urinary phosphate).

## Results

### Study population

The two study groups are depicted in Fig. 1. In Group 1, we included 23 XLH children treated with burosumab only, 6 boys and 17 girls, aged between 1.9 and 15.6 years at the initiation of burosumab therapy. Four participants were pubertal (Tanner stage II 1/4 and III 3/4) at the start of burosumab (B0). Patients from Group 1 received the conventional therapy for an average duration of 6.1 years (min. 1.8/max. 15.1). Biochemical and auxological parameters used in the follow-up of XLH are presented for the prepubertal and pubertal subgroups, at the start of burosumab (B0) and after one year of burosumab (B1) in Table 1 and Additional file 1 Table S1.

**Table 1:** Auxological parameters for group 1 (burosumab only) at moments of interest

Parameter (SDS)	Pre-pubertal (N = 19)		Pubertal (N = 4)		All (N = 23)	
	Mean ± SD	Min/Max	Mean ± SD	Min/Max	Mean ± SD	Min/Max
Height at B0	− 0.9 ± 1.0	− 3.2/0.9	− 1.2 ± 0.7	− 2.0/− 0.3	− 1.0 ± 1.0	− 3.2/0.9
Height at B1	− 0.9 ± 1.0	− 3.1/1.0	− 1.0 ± 0.8	− 2.0/0.0	− 0.9 ± 0.9	− 3.1/1.0
BMI at B0	1.0 ± 0.9	− 0.4/3.1	1.4 ± 0.9	0.6/2.8	1.0 ± 0.9	− 0.4/3.1
BMI at B1	1.0 ± 1.1	− 0.4/3.3	2.5 ± 2.3	0.9/6.0	1.3 ± 1.4	− 0.4/6.0
∆H B1–B0	0.0 ± 0.3	− 0.6/0.9	0.1 ± 0.3	− 0.2/0.5	0.0 ± 0.3	− 0.6/0.9
∆BMI B1–B0	0.0 ± 0.4	− 1.2/0.7	1.1 ± 1.3	0.3/3.2	0.2 ± 0.7	− 1.2/3.2

Mean ± SD, minimum and maximum for auxological parameters

B0: initiation of burosumab therapy, B1: 1 year after burosumab initiation, BMI: body mass index

In Group 2, we included 13 children with XLH aged between 6 and 16.8 years who received rhGH for at least one year during conventional therapy, and pursued rhGH when switching to burosumab therapy; three boys and one girl were prepubertal, while two boys and seven girls were pubertal. The pubertal Tanner stages documented at the start of burosumab (B0) were as follows: II: 5, III: 3 and IV: 1. Patients from Group 2 received conventional therapy, for an average duration of 8.7 years (min. 1.2/max. 15.8). Growth and biochemical parameters of interest for the follow-up of XLH are shown separately for the prepubertal and pubertal subgroups in Table 2 and Additional file 2: Table S2, respectively.

**Table 2:** Auxological parametersf or group 2 (burosumab and rhGH) at moments of interest

Parameter (SDS)	Pre-pubertal (N = 4)		Pubertal (N = 9)		All (N = 13)	
	Mean ± SD	Min/Max	Mean ± SD	Min/Max	Mean ± SD	Min/Max
Height at GH0	− 2.1 ± 0.5	− 2.8/− 1.6	− 2.1 ± 0.6	− 3.3/− 1.2	− 2.1 ± 0.5	− 3.3/− 1.2
Height at GH1	− 1.4 ± 0.4	− 2.0/− 0.8	− 1.6 ± 0.8	− 3.1/− 0.5	− 1.5 ± 0.7	− 3.1/− 0.5
Height at B0	− 1.0 ± 0.4	− 1.6/− 0.6	− 1.3 ± 0.9	− 3.0/0.0	− 1.2 ± 0.8	− 3.0/0.0
Height at B1	− 0.7 ± 0.4	− 1.2/− 0.1	− 1.1 ± 0.8	− 2.5/0.2	− 0.9 ± 0.7	− 2.5/0.2
BMI at GH0	0.5 ± 0.8	− 0.5/1.6	0.1 ± 0.9	− 1.0/2.1	0.2 ± 0.9	− 1.0/2.1
BMI at GH1	0.6 ± 0.9	− 0.4/1.5	0.2 ± 0.9	− 0.8/2.0	0.3 ± 0.9	− 0.8/2.0
BMI at B0	0.7 ± 0.6	− 0.1/1.5	0.0 ± 0.7	− 1.0/1.0	0.2 ± 0.7	− 1.0/1.5
BMI at B1	0.7 ± 0.8	− 0.1/1.6	0.1 ± 0.6	− 0.7/1.0	0.3 ± 0.7	− 0.7/1.6
∆H GH1–GH0	0.7 ± 0.1	0.5/0.9	0.5 ± 0.3	0.2/1.2	0.6 ± 0.3	0.2/1.2
∆H B1–B0	0.3 ± 0.1	0.2/0.5	0.2 ± 0.2	− 0.2/0.5	0.2 ± 0.1	− 0.2/0.5
∆H B0–GH0	1.1 ± 0.1	1.0/1.2	1.0 ± 0.5	0.3/1.8	1.0 ± 0.4	0.3/1.8
∆BMI B0–GH0	0.1 ± 0.2	− 0.1/0.4	0.0 ± 0.4	− 1.1/0.5	0.0 ± 0.4	− 1.1/0.5
∆BMI B1–B0	0.0 ± 0.4	− 0.3/0.7	0.1 ± 0.2	− 0.4/0.4	0.0 ± 0.3	− 0.4/0.7

Mean ± SD, minimum and maximum for auxological parameters

B0: initiation of burosumab therapy, B1: 1 year after burosumab initiation, GH0: initiation of rhGH therapy, GH-1: 1 year before rhGH initiation, GH1: 1 year after rhGH initiation, BMI: body mass index

### Burosumab and growth

**Fig. 2:**
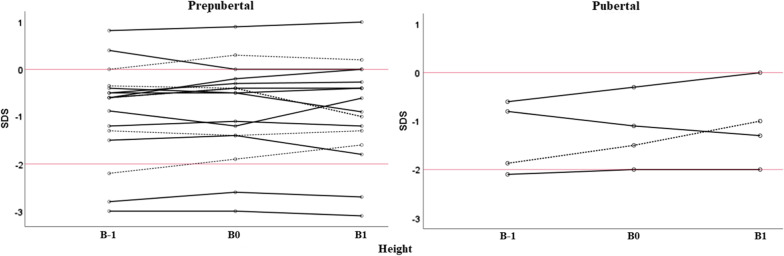
Individual changes in height SDS in group 1. Solid black lines correspond to girls and dashed black lines to boys. The solid red lines mark 0 and − 2 SDS

The change in height SDS in XLH children treated with burosumab only (group 1) for one year, i.e., ∆H SDS, is shown in Fig. 2. The means for height SDS at the start of burosumab and after one year of treatment were comparable in the prepubertal and pubertal subgroups and, on average, hardly changed after 1 year of therapy: mean ± SD ∆H B1–B0 was 0.0 ± 0.3 for the prepubertal XLH children and 0.1 ± 0.3 for the pubertal XLH adolescents (Table 1).

BMI SDS was comparable at the start and after one year of burosumab in the prepubertal subgroup: mean ± SD ∆BMI 0.0 ± 0.4. Interestingly, the pubertal adolescents with XLH increased their BMI between the start and the evaluation at one year of therapy: mean ± SD ∆BMI 1.1 ± 1.3 (see Table 1).

### Burosumab and rhGH therapy

The median ages at the time of starting rhGH and burosumab therapies were 5.7 and 7.7 years, respectively. The means ± SD for the time, in years, between the initiation of rhGH (GH0) and the initiation of burosumab (B0) were 1.7 ± 0.6 and 3.3 ± 2.4 in the prepubertal and pubertal subgroups, respectively.

**Fig. 3:**
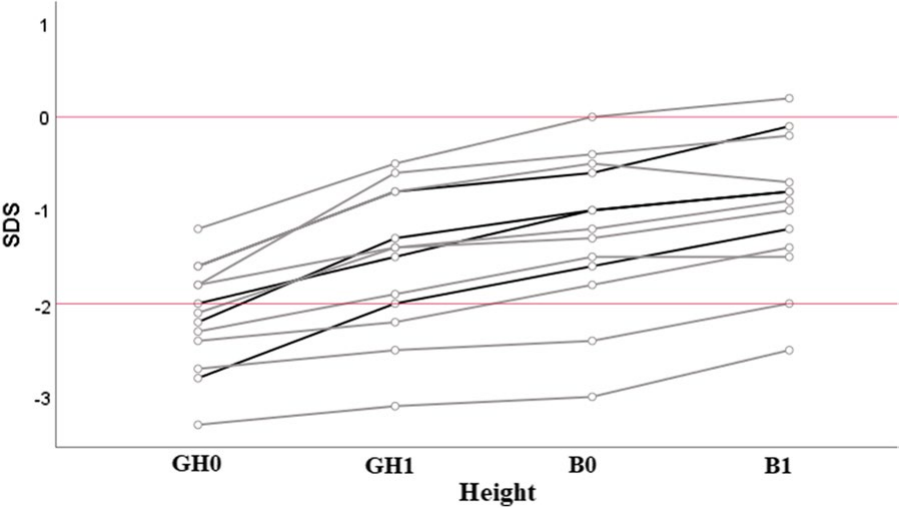
Individual changes in height SDS in group 2. Black and grey lines correspond to prepubertal and pubertal participants, respectively. The solid red lines mark 0 and − 2 SDS

The change in height SDS after the initiation of rhGH therapy and its progression under conventional therapy and burosumab are shown in Fig. 3 and in Table 2. The largest gain in height SDS was obtained during the first year of rhGH therapy and maintained thereafter during burosumab therapy.

**Fig. 4:**
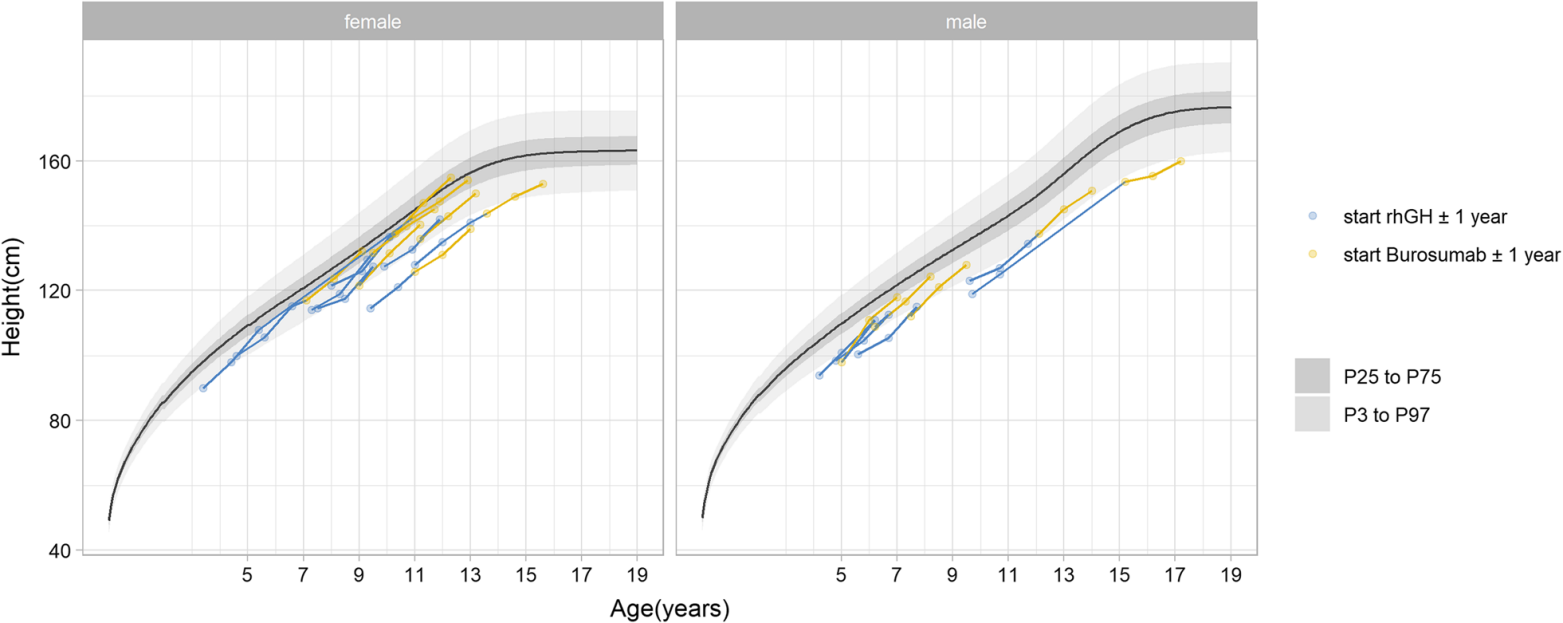
Individual growth pattern in patients treated with rhGH and burosumab. P: percentile

The means ± SD for height SDS at initiation of burosumab (B0) and after one year of concomitant treatment of burosumab and rhGH (B1) were comparable in the prepubertal and pubertal subgroups and improved slightly (for the whole group − 1.2 ± 0.8 to − 0.9 ± 0.7) between B0 and B1 (see Table 2). When burosumab was initiated, both prepubertal and pubertal populations showed an improved height SDS, compared to the point at which rhGH therapy had been initiated (mean ± SD prepubertal: − 2.1 ± 0.5 at GH0 vs. − 1.0 ± 0.4 at B0, pubertal: − 2.1 ± 0.6 at GH0 vs. − 1.3 ± 0.9 at B0). To visualize individual growth in this group, we plotted the measured height values in centimeters on the WHO growth curves (Fig. 4).

RhGH therapy improved height before the initiation of burosumab (mean ± SD ∆H B0–GH0 1.0 ± 0.4 for the entire group 2). The mean difference in height SDS after 1 year of burosumab and rhGH therapy was found to be 0.2 ± 0.1 SDS.

BMI SDS did not vary after burosumab initiation in the prepubertal and pubertal subgroups (Table 2).

### Comparison of height change after 1 year of burosumab therapy between the two study groups

After adjusting for pubertal status and height SDS at the start of burosumab, we compared the gain in height during the first year of burosumab therapy, i.e., ∆H B1–B0, between the two main groups. The resulting difference in least squares means of 0.18 SDS (95% CI − 0.05 bis 0.41, p = 0.127) between children treated with burosumab only and children treated with burosumab and rhGH was not statistically significant. We then looked at height change in SDS between B1 and B0 within each group: the least square mean change in children treated with burosumab only was 0.04 SDS (95% CI − 0.1 to + 0.18, p = 0.534), and not statistically significant, while in children who received the combination of burosumab and rhGH, there was a significant change of 0.22 SDS (95% CI 0.06 to 0.39; p = 0.009).

### Biochemistry and safety

**Fig. 5:**
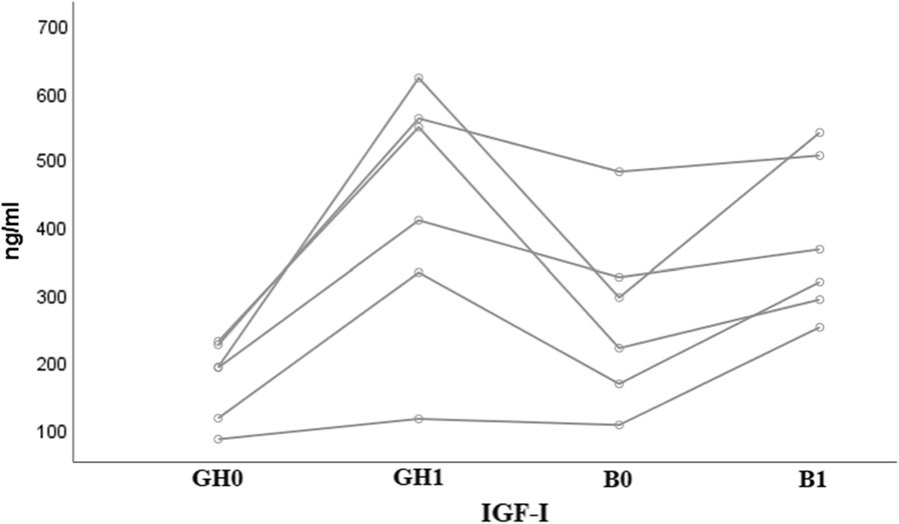
Distribution of serum IGF-I levels in the pubertal group 2 population. Normal ranges depended on the Tanner stage. II: 198–551 ng/ml, III: 238–672 ng/ml, IV: 318–870 ng/ml, V: 302–774 ng/ml

RhGH doses at B0 and B1 were comparable in the two subgroups (prepubertal and pubertal) and they did not exceed 70 μg/kg/day (see Additional file 1: Table S2). IGF-I levels were documented as raw data, showing higher levels in the pubertal subgroup after one year of rhGH and conventional therapy (GH1) and after one year of burosumab and rhGH therapy (B1), but all values remained in the normal range for age and pubertal stage (Fig. 5). Due to a high rate of missing values, a similar analysis was not possible for the prepubertal group.

**Fig. 6:**
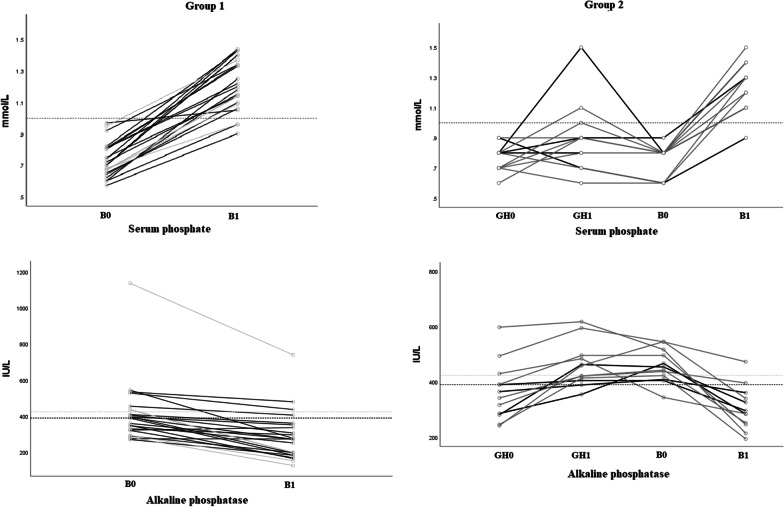
Individual changes in serum phosphate and alkaline phosphatase. Black and grey lines correspond to prepubertal and pubertal participants, respectively. Dashed lines show the lower limit of normal for serum phosphate (1.0–1.85 mmol/L) and the upper limit of normal for alkaline phosphatase: in black and grey for the prepubertal (50–390 IU/L) and pubertal (131–424 IU/L) populations, respectively

**Fig. 7:**
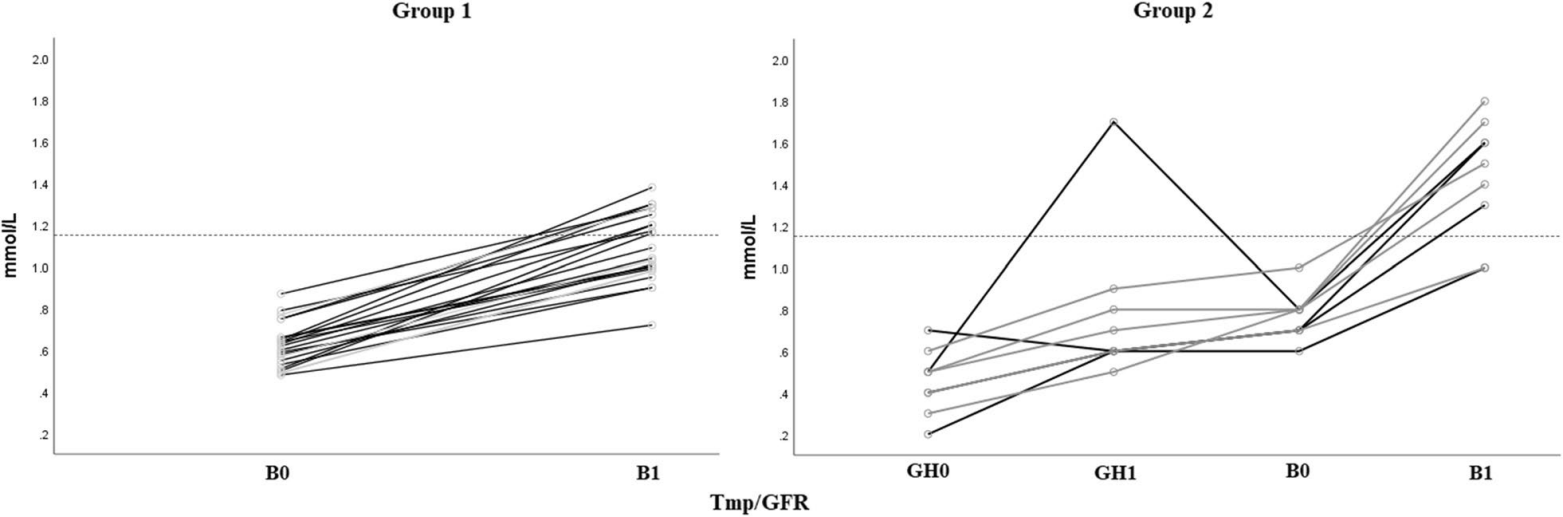
Individual changes in TmP/GFR. Black and grey lines correspond to prepubertal and pubertal participants, respectively. Dashed black lines show the lower limit of normal (1.15–2.44 mmol/L)

The distributions of serum phosphate and alkaline phosphatase levels are plotted in Fig. 6. Most participants showed higher serum phosphate levels after 1 year of rhGH therapy, but these were, with one exception, still lower than the normal range; at the start of burosumab, which corresponds to an average of 2.8 years (min. 1/max. 7.7 years) after starting rhGH treatment, all values were clearly below the lower limit of the normal range. As expected, after 1 year of burosumab therapy, the upward trend in fasting serum phosphate levels is evident for all patients treated with burosumab in Fig. 6. None of the patients in either group showed hyperphosphatemia. This is consistent with the increase in TmP/GFR values over time in both groups receiving burosumab (Fig. 7). Alkaline phosphatase normalized in most patients from both groups during burosumab therapy. There was no change in serum calcium levels or urinary calcium excretion. There was no noticeable change in 25-OHD levels, but 1,25-(OH)_2_D increased dramatically under burosumab in both the prepubertal and pubertal subgroups, regardless of whether they received rhGH. Median values for PTH are shown in Additional file 1: Table S1 and Additional file 2: Table S2.

## Discussion

Short stature is a major feature of XLH and is severe and inevitable if the disease is left untreated [12, 13, 30], and affects most patients treated with a combination of phosphate supplements and vitamin D analogs, i.e., conventional therapy [31–34]. Novel therapies that antagonize the systemic FGF23 excess in XLH children significantly improve rickets and osteomalacia, but do not have a dramatic effect on growth velocity in clinical trials and reports [25–29, 35]. As physicians we will not be able to produce in the short- to mid-term data on final height in patients treated with FGF23 blockers. In this context, we felt it important to report the efficacy and safety of burosumab alone or in combination with rhGH in our prospective observational cohort of XLH children.

Reports in the literature describe different values for changes in height SDS when rhGH therapy is added to conventional therapy in children with XLH, especially before puberty. The rhGH doses used for treatment and the follow-up periods also vary between studies. In a previous study, we found that rhGH improves height SDS in both prepubertal and pubertal children with XLH after 1 year of treatment, with a stronger impact in prepubertal children [19]. Our descriptive analysis of growth after the initiation of rhGH therapy while patients were under conventional therapy confirms this initial height gain in this novel study. Indeed, during our follow-up of growth, the participants gained a median of 0.6 SDS in height in 1 year, and a median total increase of 1 SDS at the time burosumab was initiated. This gain in height SDS is comparable to that found in other published studies. Mäkitie et al. reported a similar change in height SDS (0.1–0.7 SDS) in 10 children treated with rhGH for 12 months [16]. An even greater catch-up growth of 1.1 SDS was reported by Živičnjak et al. in 16 prepubertal patients treated with rhGH for 3 years while also on conventional therapy [14]. In our study, the documented positive effect of rhGH on growth continued through the period analyzed for the population on combined therapy, with a gain of another 0.2 SDS after the initiation of burosumab. The improvement in height SDS did gradually slow down, when compared to the first year of rhGH therapy, in accordance with previous observations made by our group and by Haffner et al. [19, 20, 23]. It has also been shown that after discontinuing rhGH there may be catch-down growth [21], and hence, also based on our data, continuing treatment with rhGH after switching the conventional therapy to burosumab might be beneficial for the final height. Few studies on the final height after rhGH therapy have been performed and the duration of the administration is very variable. While Baroncelli et al. [24] and Haffner et al. [23] reported a positive effect of rhGH therapy and a final height that exceeds the predicted genetic height, Meyerhoff et al. [22] concluded that 3 years of rhGH therapy led to a statistically nonsignificant gain of 0.7 SDS when compared to the baseline.

In our cohort, rhGH therapy did not induce an increase in IGF-I above the normal range. This result is in accordance with that of Seikaly et al. [21], but contrasts with some other reports, where different rhGH doses were used [14, 20].

Levels of serum phosphate increased transiently after rhGH initiation in our study population, but at the time of switching the therapeutic regime to burosumab, all participants had values below 1 mmol/L. This documented increase in the first year of rhGH therapy is consistent with the literature [14, 16, 24]. Interestingly, we did not observe a rebound effect in patients treated with rhGH when switched to burosumab; none of them experienced hyperphosphatemia. Further, TmP/GFR remained in the low normal range for all patients during rhGH. Vitamin D and urinary calcium excretion also remained unchanged in patients on conventional therapy and after 1 year of rhGH therapy (data not shown).

Because the first clinical trial reporting the use of burosumab for the treatment of XLH in children was published in 2018 [25], there is a paucity of data on height and growth under burosumab therapy. Reported changes in height SDS in prepubertal children were 0.15 ± 0.04 SDS in the study by Carpenter et al. [25], 0.17 SDS in the study by Imel et al. [27] and 0.15 to 0.17 SDS in the recent study from Ward et al. [29]. Unlike the study of Imel et al. [27], our data showed no significant change in height SDS in patients who received burosumab only. Even though our sample is comparable in size with that of Imel et al. [27], our calculated least squares mean was 0.04 SDS at 12 months, after adjusting for pubertal status and height at the time that burosumab was initiated. One possible explanation for the contrasting results is the different observation period, as the calculated value reported by Imel et al. is only for the 64-week time point. Further, it is unclear if an adjustment of the statistical analysis for pubertal stage was done. These differences might be caused by the multicenter nature of the trial, hence height measurements were made in different locations and likely using different devices; in our study, all measurements were made in the same clinic, using the same device and by the same investigator. Ramos et al. [35] concluded that burosumab alone has a minimal to no effect on growth, in accordance with our findings. Our results are comparable with a recent study from Ward et al. [29]; the means of least square changes in height SDS under burosumab therapy were basically the same, but our analysis, when adjusting for pubertal stage, showed that these differences are not statistically significant. We conclude from these observations that, in children with XLH, despite the near-normalized serum phosphate levels, no major gain in height SDS is attained after 1 year of burosumab therapy. These findings further suggest that the blockade of the systemic effects of the FGF23 excess does not fully overcome the growth impairment. Other biochemical or molecular defects, likely located in the growth plate, participate in growth impairment in XLH [36].

In contrast to the lack of height SDS improvement in XLH children who received only burosumab, we report here a statistically significant change in height SDS after 1 year of burosumab therapy in XLH children who received the combined burosumab and rhGH therapy. In addition to the catch-up growth that followed the initiation of rhGH therapy, height SDS changed by 0.22 SDS in the first year after the initiation of burosumab. Our data suggest that the addition of rhGH to burosumab is beneficial for the growth of XLH children. Interestingly, this observation in XLH children echoes the observations in *Hyp* mice reported by Fuente et al. who demonstrated that inhibition of the FGF receptor pathway in young *Hyp* mice had a synergistic effect with growth hormone on skeletal growth and growth plate structure [11].

The patients reported here belong to one of the largest cohorts of XLH children in Europe; most of these patients have contributed to different reports on burosumab-related outcomes [28, 37–39]. To our knowledge, this is the first study on growth under concomitant rhGH and burosumab therapy. We considered it important to report our experience with this combination of therapies in order to evaluate both the efficacy and the safety of these treatments. Indeed, our rhGH-treated study population was followed up for a relatively long time, most of the participants receiving rhGH for more than 2 years; in addition, the number of children reported here is rather large when we consider the rarity of the disease and the treatment.

Due to the small sample sizes in most of the pediatric studies that have analyzed growth in XLH (treated with conventional therapy and rhGH or after switching to burosumab), including ours, and as expected in the context of rare diseases, it is important to interpret the results with caution. As already mentioned, we adjusted our statistical analysis for parameters that are important for deciding whether rhGH therapy should be given. As this is an individual treatment decision in XLH-related short stature, based on height and pubertal status, and as it does not depend on the causal treatment regimes, it was necessary to adjust the statistical analysis for these parameters. In our study we tried to assess effects and differences not only regarding statistical significance, but also regarding clinical relevance, the latter being even more important than the former [3, 4, 40]. In particular, statistically nonsignificant results in our sample do not necessarily translate into inexistent clinically relevant effects in the population, because of the restricted sample size in this rare disease. Our strategy was to report p-values for statistical contrasts corresponding to primary research questions and to deliver the remaining exploratory results without p-values. However, all effect estimates are provided with confidence intervals that are known to indicate statistical significance. This is in line with the guidelines for reporting statistical results of, for example, the New England Journal of Medicine [41, 42].

We recognize several limitations in our report. A group of patients receiving both rhGH and burosumab for the same amount of time would have been an optimal population for studying their combined effect on growth, but we have not been able to analyze such a group, rhGH therapy not always being started at the same time as burosumab.

If it were ethical and feasible to randomize treatments in a sufficiently large XLH cohort, this would deliver the most reliable results. Since only observational data are available, a fair comparison between various ways of treatment would necessitate adjustment for all potential confounders in the treatment comparison (e.g., by propensity score methods). These methods, however, need larger datasets than ours, which remains a real difficulty when reporting on rare diseases. Therefore, we chose to approximate this optimal analysis in our observational study by way of an adjustment of the treatment comparison for a small number of important and available variables. We considered the two groups of XLH children to be comparable at the start of burosumab, concerning the potential effect of burosumab on growth, even though the children of group 2 consisted of pre-selected individuals who had previously received rhGH due to short stature. The characteristics of both populations for analysis (height, age, biochemical profile) were similar at the start of burosumab.

## Conclusion

We found that burosumab therapy alone does not have any visible effect on height SDS, but that rhGH therapy led to catch-up growth during conventional therapy, as expected, an effect that was maintained throughout when patients were switched to burosumab therapy. It is important to report the absence of hyperphosphatemia and safety issues in children who received the combination of burosumab and rhGH. To further understand and show the effect of rhGH in combination with burosumab, we need data on patients starting on rhGH during burosumab therapy. However, studies with longer periods of observation are required.

## Methodology

### Study design and population, auxological and biochemical measurements

Since March 1st, 2018, all children diagnosed with XLH and treated with burosumab are included in a prospective longitudinal single-center cohort at the French National Reference Center for Rare Disorders of the Calcium and Phosphate Metabolism, Filière OSCAR and Platform of expertise for rare diseases, ENDO-ERN and ERN-BOND, Bicêtre Paris-Saclay Hospital, France. The cohort study is registered with ClinicalTrials.gov (identifier NCT04419363, https://clinicaltrials.gov/ct2/show/NCT04419363). Its main purpose was of having a prospective longitudinal cohort of children treated with burosumab, and to gather important auxological and biochemical data for future studies.

At the date of study, i.e., Dec 31st, 2021, among the 60 children already included in the cohort, 36 had completed at least 1 year of burosumab therapy at the time of the analysis and were included in this observational analysis. Data collected for this specific study were processed in a pseudonymized format. All patients initially received conventional therapy with vitamin D analogs and phosphate supplements, in accordance with international guidelines (2). In accordance with the French indication for burosumab, all patients were eventually switched from conventional therapy to burosumab. We then distinguished the following two groups: group 1 consisted of 23 individuals who received only burosumab, i.e., they were “rhGH naïve” (Fig. 1); these patients either did not present short stature or declined the offer of rhGH in combination with conventional therapy. Group 2 consisted of 13 participants who received rhGH for short stature, initiated during conventional therapy due to severe short stature, as an attempt to improve final height. They received rhGH for at least 1 year during conventional therapy and for at least 1 year after the initiation of burosumab. The therapy with rhGH was not stopped at the moment of burosumab initiation, as we considered the patients might potentially benefit future, decision based on our own published data [19] and on the centers experience with rhGH in XLH. Further, based on pubertal Tanner stage at the initiation of burosumab therapy, we analyzed height changes in prepubertal and pubertal children, respectively. Four other patients from the original cohort were given rhGH only after burosumab had been initiated; because they were so few, they were not included in this analysis. For this manuscript, we only considered patients who had received rhGH before switching from conventional therapy to burosumab and continued rhGH after the switch to burosumab for the treatment of XLH.

Auxological (height, body mass index [BMI]) and biochemical data were gathered from medical charts for the key time points defined as: GH0, initiation of rhGH therapy (Group 2), GH1, 1 year after initiation of rhGH therapy (Group 2), B0, initiation of burosumab (Group 1 and 2), and B1, 1 year after burosumab initiation (Groups 1 and 2). Additionally, height was also documented 1 year before these time points (GH-1 and B-1) (Fig. 1). The participants did not present with any other health problems that might influence growth (e.g., chronic systemic diseases or inflammatory intestinal disorders).

All participants had their height and weight measured at the follow-up visits to our clinic, at least twice a year during conventional therapy, and every 3 months during burosumab therapy. For the measurement of height, we used a Harpenden stadiometer. These parameters, as well as calculated BMI, were analyzed as standard deviation scores, using WHO reference data for height and BMI [43]. Delta height (∆H) parameters were also calculated as follows: Group 1: ∆H B1–B0 for the difference in height SDS after 1 year of burosumab therapy, Group 2: ∆H GH1–GH0 for the difference in height SDS after 1 year of rhGH therapy during conventional therapy, ∆H B1–B0 for the difference in height SDS after 1 year of burosumab combined with rhGH, and ∆H B0–GH0 for the difference in height SDS between the initiation of burosumab and the start of rhGH. Intra-individual change in BMI-SDS (∆BMI) was analyzed in the same way as for ∆H.

Parental height and genetic target height were documented for all patients, but not included in the analysis due to the high proportion of familial XLH cases in the study population, which might have biased the interpretation.

We had also planned to gather data on bone age at the start of burosumab (B0) and after 1 year of treatment (B1), but on the one hand, the severity of rickets on some radiographs hindered analysis of bone age, and on the other hand, we felt there were too many missing data at these time points. For these reasons, we decided to exclude this parameter.

Routine biochemical tests were performed, either in our hospital at the follow-up visit or in designated external laboratories. The parameters documented were the following: levels of serum phosphate, alkaline phosphatase, calcium, creatinine, 25-hydroxyvitamin D (25-OHD), 1,25-dihydroxyvitamin D (1,25-(OH)_2_D), parathyroid hormone (PTH), urinary calcium excretion (urinary calcium/creatinine ratio), phosphate reabsorption (tubular reabsorption of phosphate (TRP), and renal tubular maximum reabsorption rate of phosphate to glomerular filtration rate (TmP/GFR]). For patients treated with rhGH (Group 2), serum insulin-like growth factor-I (IGF-I) levels were measured to analyze the safety of rhGH therapy; for the analysis, we considered only values from the time of the visit or no more than 6 months earlier. The dose of rhGH was regularly adjusted in this group, based on growth velocity, weight and serum IGF-I levels. The target was an IGF-I in the normal range for age, gender, and pubertal stage, when compared to reference population data provided by the assay’s manufacturer.

### Statistical analysis

The descriptive analysis of the study sample was performed using IBM SPSS Statistics 25.0 software. The continuous variables with a normal distribution are described using the mean, SD, and range (minimum and maximum) and those with asymmetric distributions are shown using the median and range. Height in centimeters was plotted on WHO growth curves [43] for each participant in group 2, using the documented values at the moments of interest (GH-1, GH0, GH1, B-1, B0, B1).

For the comparison of ∆H SDS between the two groups and within the groups, we used an analysis of the covariance model (PROC GLM in SAS 9.4) to adjust for pubertal stage, as well as for height SDS at B0 (initiation of burosumab). Least squares means and mean differences were estimated from this model with 95% confidence intervals. P-values below 0.05 are considered to indicate statistical significance.

## Supplementary Information

**Additional file 1: Table S1.** Demographic, clinical and biochemical characteristics for group 1 (burosumab only). Median, minimum and maximum for demographic and biochemical parameters.

**Additional file 2: Table S2** Demographic, clinical and biochemical characteristics for group 2 (burosumab and rhGH). Median, minimum and maximum for demographic and biochemical parameters

## Data Availability

The datasets used and/or analyzed during the current study are available from. the corresponding author upon reasonable request.

## Abbreviations

BMI: Body mass index
CI: Confidence interval
FGF23: Fibroblast growth factor 23
IGF-I: Insulin-like growth factor I
PTH: Parathyroid hormone
rhGH: Recombinant human growth hormone
SDS: Standard deviation score
urinary Ca/Creat: Urinary calcium/creatinine ratio
TmP/GFR: Ratio of renal tubular maximum reabsorption rate of phosphate to glomerular filtration rate
TRP: Tubular reabsorption of phosphate
WHO: World Health Organization
XLH: X-linked hypophosphatemia
25-OHD: 25-Hydroxyvitamin D
1,25-(OH)_2_D: 1,25-Dihydroxyvitamin D
B-1: 1 Year before burosumab initiation
B0: Initiation of burosumab
B1: 1 Year after burosumab initiation
GH-1: 1 Year before initiation of rhGH
GH0: Initiation of rhGH
GH1: 1 Year after initiation of rhGH
∆H: Delta height (difference in height between two given moments)
∆H B1–B0: Difference in height after 1 year of burosumab therapy
∆H GH1–GH0: Difference in height after 1 year of GH therapy
∆H B0–GH0: Difference in height between initiation of rhGH and initiation of burosumab
∆BMI: Delta BMI (difference in BMI between two given moments)
