# Supplementary material for: Growth pattern in children with X-linked hypophosphatemia treated with burosumab and growth hormone
Source: Orphanet J Rare Dis. 2022 Nov 12;17:412. doi: 10.1186/s13023-022-02562-9 (PMC9652849; doi:10.1186/s13023-022-02562-9)
Supplement: Supplementary file 1 — Additional file 1: Table S1. Demographic, clinical and biochemical characteristics for group 1 (burosumab only). Median, minimum and maximum for demographic and biochemical parameters. [file 13023_2022_2562_MOESM1_ESM.docx]

| **Paramater (unit)** | **Prepubertal (N=19)** | | **Pubertal (N=4)** | | **All (N=23)** | |
| --- | --- | --- | --- | --- | --- | --- |
|  | ***Male: 5***  ***Female: 14*** | | ***1***  ***3*** | | ***6***  ***17*** | |
|  | **Median** | **Min/Max** | **Median** | **Min/Max** | **Median** | **Min/Max** |
| Age at start conventional treatment (years) | 0.9 | 0.1/6.0 | 1.7 | 0.3/9.0 | 0.9 | 0.1/9.0 |
| Age at B0 (years) | 7.6 | 1.9/9.7 | 12.4 | 11.4/15.6 | 7.8 | 1.9/15.6 |
| Burosumab dose at B1 (mg/kg/dose) | 1.2 | 0.8/2.0 | 1.3 | 0.4/2.0 | 1.2 | 0.4/2.0 |
| PTH B0 (pg/ml)* | 33.0 | 18.0/96.0 | 60.0 | 34.0/94.0 | 34.0 | 18.0/96.0 |
| PTH B1 (pg/ml)* | 53.0 | 17.0/118.0 | 68.0 | 42.0/119.0 | 54.0 | 17.0/119.0 |
| 25-OHD B0 (ng/ml)* | 33.5 | 15.0/79.0 | 35.5 | 28.0/44.0 | 34.0 | 15.0/79.0 |
| 25-OHD B1 (ng/ml)* | 32.0 | 20.0/64.0 | 25.5 | 14.0/34.0 | 31.0 | 14.0/64.0 |
| 1,25-(OH)_2_D B0 (pg/ml)* | 17.0 | 7.0/29.0 | 39.0 | 26.0/55.0 | 22.0 | 7.0/55.0 |
| 1,25-(OH) _2_D B1 (pg/ml)* | 65.0 | 21.0/86.0 | 88.0 | 62.0/119.0 | 65.5 | 21.0/119.0 |
| ALP B0 (IU/L)* | 389.0 | 271.0/544.0 | 364.5 | 283.0/1136.0 | 389.0 | 271.0/1136.0 |
| ALP B1 (IU/L)* | 274.0 | 166.0/480.0 | 176.5 | 127.0/740.0 | 273.0 | 127.0/740.0 |
| Serum phosphate B0 (mmol/L)* | 0.7 | 0.6/1.0 | 0.6 | 0.7/1.0 | 0.7 | 0.6/1.0 |
| Serum phosphate B1 (mmol/L)* | 1.2 | 0.9/1.4 | 1.2 | 1.0/1.4 | 1.1 | 0.9/1.4 |
| Serum calcium B0 (mmol/L)* | 2.4 | 2.3/2.8 | 2.4 | 2.4/2.5 | 2.4 | 2.3/2.8 |
| Serum calcium B1 (mmol/L)* | 2.5 | 2.4/2.7 | 2.3 | 2.4/2.4 | 2.5 | 2.4/2.7 |
| Urinary Ca/Crea B0 (mmol/mmol)* | 0.4 | 0.1/0.9 | 0.3 | 0.3/0.4 | 0.3 | 0.1/0.9 |
| Urinary Ca/Crea B1 (mmol/mmol)* | 0.3 | 0.1/0.8 | 0.2 | 0.1/0.5 | 0.3 | 0.1/0.8 |
| TRP B0 (%) | 84.1 | 61.0/94.5 | 80.0 | 74.0/85.5 | 83.3 | 61.0/94.5 |
| TRP B1 (%) | 90.3 | 83.0/96.2 | 89.9 | 86.7/93.5 | 90.1 | 83.0/96.2 |
| TmP/GFR B0 (mmol/L)* | 0.6 | 0.5/0.9 | 0.5 | 0.5/0.8 | 0.6 | 0.5/0.9 |
| TmP/GFR B1 (mmol/L)* | 1.0 | 0.7/1.4 | 1.0 | 1.0/1.3 | 1.0 | 0.7/1.4 |

**Normal range : PTH 18.5- 88 pg/ml, 25-OHD 30- 100 ng/ml, 1,25-(OH)_2_D 20-70 pg/ml, alkaline phosphatase : prepubertal 50- 390 IU/L, pubertal 131-424 IU/L, serum phosphate 1- 1.85 mmol/L, serum calcium 2.2- 2.7 mmol/L, urinary calcium/creatinine (mmol/mmol) : 1-3 y.o. <1.4, 3-5 y.o. <1.1, 5-7 y.o. <0.8, >7 y.o. <0,7, TmP/GFR: 1.15-2.44 mmol/L*
