# Supplementary material for: Growth pattern in children with X-linked hypophosphatemia treated with burosumab and growth hormone
Source: Orphanet J Rare Dis. 2022 Nov 12;17:412. doi: 10.1186/s13023-022-02562-9 (PMC9652849; doi:10.1186/s13023-022-02562-9)
Supplement: Supplementary file 2 — Additional file 2: Table S2 Demographic, clinical and biochemical characteristics for group 2 (burosumab and rhGH). Median, minimum and maximum for demographic and biochemical parameters [file 13023_2022_2562_MOESM2_ESM.doc]

| **Parameter (unit)** | **Prepubertal (N=4)** | | **Pubertal (N=9)** | | **All (N=13)** | |
| --- | --- | --- | --- | --- | --- | --- |
|  | ***Male: 3***  ***Female: 1*** | | ***2***  ***7*** | | ***5***  ***8*** | |
|  | **Median** | **Min/Max** | **Median** | **Min/Max** | **Median** | **Min/Max** |
| Age at start conventional treatment (years) | 1.1 | 0.3/1.8 | 1.5 | 1.0/3.0 | 1.5 | 0.3/3.0 |
| Age at GH0 (years) | 5.7 | 5.0/6.7 | 9.4 | 4.4/12.0 | 8.5 | 4.4/12.0 |
| Age at B0 (years) | 7.7 | 6.0/8.5 | 12.0 | 10.1/16.8 | 11.3 | 6.0/16.8 |
| Burosumab dose at B1 (mg/kg/dose) | 1.2 | 0.9/1.7 | 1.1 | 0.6/2.0 | 1.1 | 0.6/2.0 |
| RhGH dose B0 (μg/kg/d) | 44.5 | 40.0/64.0 | 58.5 | 45.0/69.0 | 51.0 | 40.0/69.0 |
| RhGH dose B1 (μg/kg/d) | 41.5 | 35.0/56.0 | 49.5 | 38.0/60.0 | 48.5 | 35.0/60.0 |
| PTH B0 (pg/ml)* | 27 | 18.1/39.0 | 48 | 24.0/171.0 | 36 | 18.0/70.0 |
| PTH B1 (pg/ml)* | 48 | 19.3/58.0 | 71 | 36.0/122.0 | 58.0 | 19.3/122.0 |
| 25-OHD B0 (ng/ml)* | 31.6 | 22.0/70.0 | 35.0 | 17.0/55.0 | 32.0 | 17.0/70.0 |
| 25-OHD B1 (ng/ml)* | 31.1 | 20.0/38.0 | 26.0 | 11.0/36.0 | 26.0 | 11.0/38.0 |
| 1,25-(OH)_2_D B0 (pg/ml)* | 18.0 | 6.0/18.0 | 48.0 | 24.0/82.0 | 28.0 | 6.0/82.0 |
| 1,25-(OH)_2_D B1 (pg/ml)* | 62.0 | 61.0/69.0 | 81.0 | 54.0/103.0 | 75.0 | 54.0/103.0 |
| ALP B0 (IU/l)* | 431.5 | 405.0/467.0 | 443.0 | 278.0/546.0 | 443.0 | 278.0/546.0 |
| ALP B1 (IU/l)* | 311.0 | 284.0/361.0 | 252.0 | 194.0/473.0 | 285.0 | 194.0/473.0 |
| Serum phosphate B0 (mmol/l)* | 0.8 | 0.6/0.9 | 0.8 | 0.4/0.8 | 0.8 | 0.4/0.9 |
| Serum phosphate B1 (mmol/l)* | 1.2 | 0.9/1.4 | 1.2 | 0.9/1.5 | 1.2 | 0.9/1.5 |
| Serum calcium B0 (mmol/l)* | 2.4 | 2.4/2.6 | 2.4 | 2.3/2.5 | 2.4 | 2.3/2.6 |
| Serum calcium B1 (mmol/l)* | 2.5 | 2.5/2.5 | 2.5 | 2.4/2.6 | 2.5 | 2.4/2.6 |
| Urinary Ca/Crea B0 (mmol/mmol)* | 0.6 | 0.2/0.7 | 0.3 | 0.1/0.6 | 0.3 | 0.1/0.7 |
| Urinary Ca/Crea B1 (mmol/mmol)* | 0.3 | 0.1/1.2 | 0.3 | 0.1/1.0 | 0.3 | 0.1/1.2 |
| TRP B0 (%) | 85.8 | 79.5/88.6 | 90.5 | 79.4/95.3 | 89.0 | 79.4/95.3 |
| TRP B1 (%) | 94.1 | 93.1/95.0 | 94.7 | 83.9/95.4 | 94.5 | 83.9/95.4 |
| TmP/GFR B0 (mmol/L)* | 0.7 | 0.6/0.8 | 0.8 | 0.3/1.0 | 0.7 | 0.3/1.0 |
| TmP/GFR B1 (mmol/L)* | 1.4 | 1.0/1.6 | 1.4 | 0.7/1.8 | 1.4 | 0.7/1.8 |

**Normal range: PTH 18.5- 88 pg/ml, 25-OHD 30- 100 ng/ml, 1,25-(OH)_2_D 20-70 pg/ml, alkaline phosphatase: prepubertal 50- 390 IU/L, pubertal 131-424 IU/L, serum phosphate 1- 1.85 mmol/L, serum calcium 2.2- 2.7 mmol/L, urinary calcium/creatinine (mmol/mmol): 1-3 y.o. <1.4, 3-5 y.o. <1.1, 5-7 y.o. <0.8, >7 y.o. <0,7, TmP/GFR: 1.15-2.44 mmol/L*
